# Correction: Retinal cholesterol metabolism is perturbated in response to experimental glaucoma in the rat

**DOI:** 10.1371/journal.pone.0281194

**Published:** 2023-01-25

**Authors:** The PLOS ONE Staff

## Notice of republication

This article was republished on January 12, 2023 to correct for errors in the Data Availability statement. The publisher apologizes for the errors. Please download this article again to view the correct version. The originally published, uncorrected article and the republished, corrected article are provided here for reference.

## Supporting information

S1 File Originally published, uncorrected article. (PDF)

S2 File Republished, corrected article. (PDF)
