# Supplementary material for: Retinal cholesterol metabolism is perturbated in response to experimental glaucoma in the rat
Source: PLoS One. 2022 Mar 11;17(3):e0264787. doi: 10.1371/journal.pone.0264787 (PMC8916636; doi:10.1371/journal.pone.0264787)
Supplement: S1 Fig — The right eye of Sprague-Dawley rats was subjected to laser photocoagulation of the trabecular meshwork, episcleral veins and limbal plexus. The left eye was considered as contralateral eye. The intraocular pressure (IOP) was monitored regularly in both eyes as well as on naive eyes (rats which were not subjected to the laser procedure), under gas anaesthesia, with a rebound tonometer (Icare®TonoLab). Results are presented as mean ± SEM of a minimum of 25 animals. Δ p<0.05, ΔΔ p<0.01, ΔΔΔ p<0.001 vs naive (unpaired t test). * p<0.05, **p<0.01, ***p<0.001 vs contralateral (paired t test). (DOCX) [file pone.0264787.s001.docx]

**
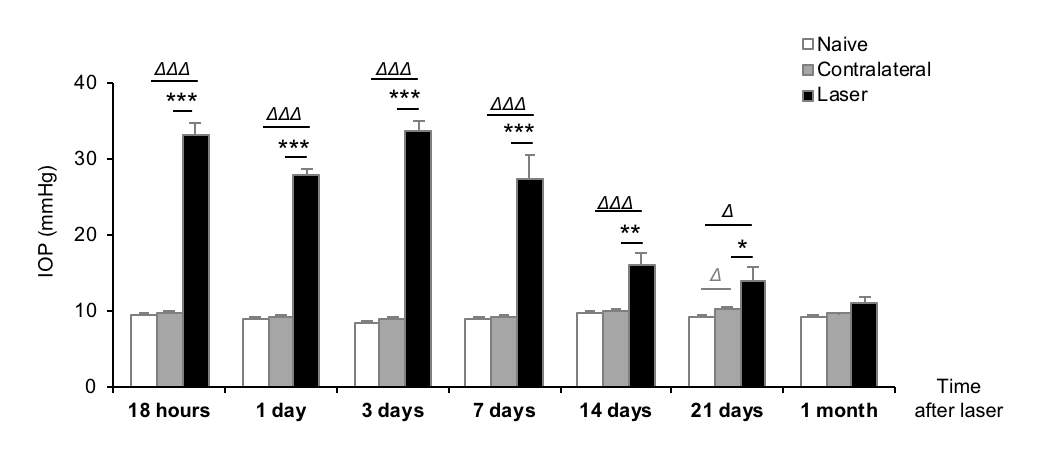
**

**S1 Fig:** **Laser procedure induces a prolonged ocular hypertension.** The right eye of Sprague-Dawley rats was subjected to laser photocoagulation of the trabecular meshwork, episcleral veins and limbal plexus. The left eye was considered as contralateral eye. The intraocular pressure (IOP) was monitored regularly in both eyes as well as on naive eyes (rats which were not subjected to the laser procedure), under gas anaesthesia, with a rebound tonometer (Icare®TonoLab). Results are presented as mean ± SEM of a minimum of 25 animals. Δ p<0.05, ΔΔ p<0.01, ΔΔΔ p<0.001 *vs* naive (unpaired t test). * p<0.05, **p<0.01, ***p<0.001 *vs* contralateral (paired t test).
