# Supplementary material for: Retinal cholesterol metabolism is perturbated in response to experimental glaucoma in the rat
Source: PLoS One. 2022 Mar 11;17(3):e0264787. doi: 10.1371/journal.pone.0264787 (PMC8916636; doi:10.1371/journal.pone.0264787)
Supplement: S2 Fig — Immunostaining for GFAP and Iba-1 was performed on retinal cryosections of naive and contralateral eyes. No major difference was observed between these two untreated groups at any time point regarding activation of macro and microglial cells under our experimental conditions. Scale bar: 50 μm. Images shown are representative of n = 6–8 retinas at 18 hours, 3 days and 1month post-laser. (DOCX) [file pone.0264787.s002.docx]

**
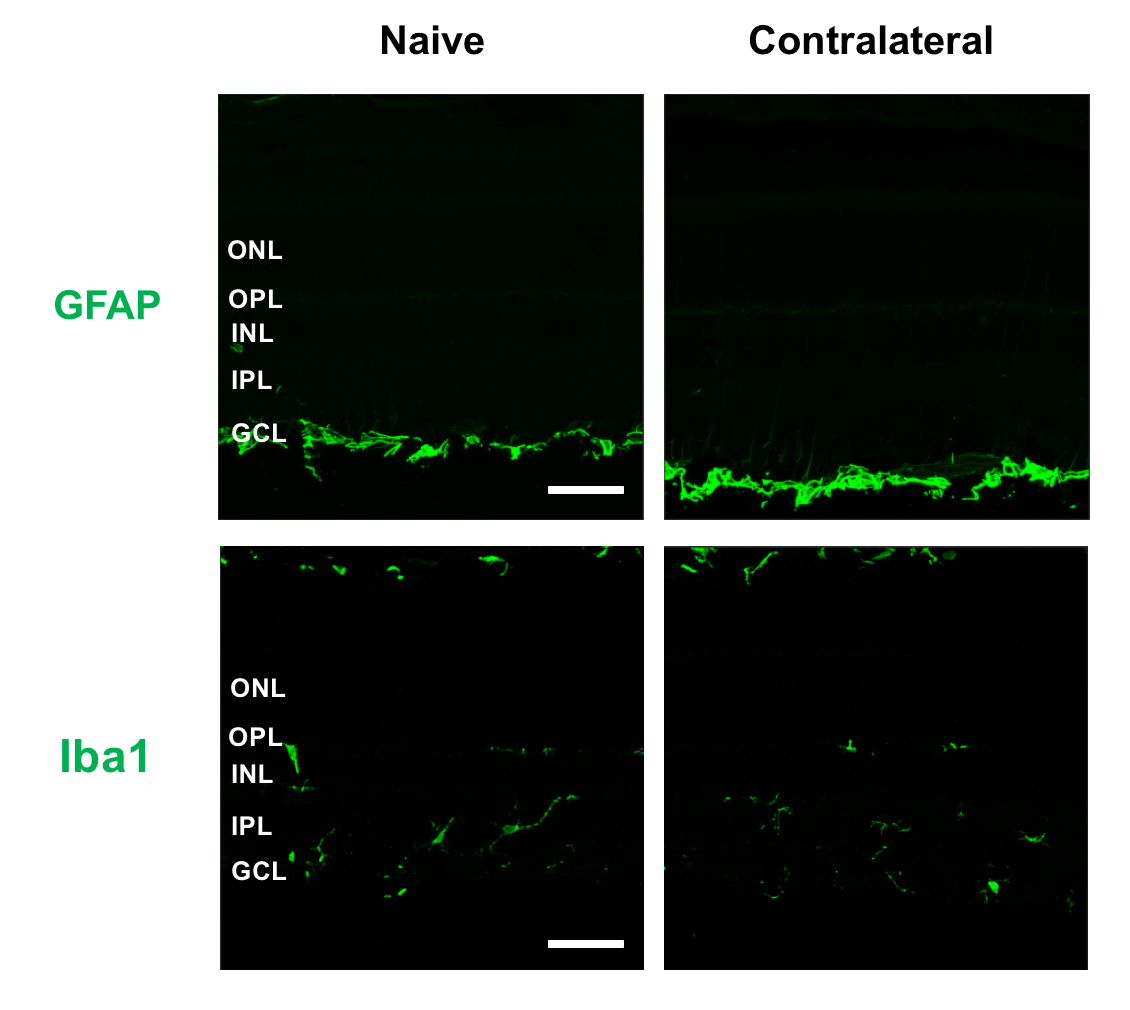
**

**S2 Fig. Glial activation was not detected in the contralateral retinas in response to laser procedure.** Immunostaining for GFAP and Iba-1 was performed on retinal cryosections of naive and contralateral eyes. No major difference was observed between these two untreated groups at any time point regarding activation of macro and microglial cells under our experimental conditions. Scale bar: 50 µm. Images shown are representative of n=6- 8 retinas at 18 hours, 3 days and 1month post-laser.
