# Supplementary material for: Retinal cholesterol metabolism is perturbated in response to experimental glaucoma in the rat
Source: PLoS One. 2022 Mar 11;17(3):e0264787. doi: 10.1371/journal.pone.0264787 (PMC8916636; doi:10.1371/journal.pone.0264787)
Supplement: S1 Table — ApoE: Apolipoprotein E, Cd68: Cluster of Differentiation 68, Cyp27a1: cytochrome P450 family 27 subfamily A member 1, Cyp46a1: cytochrome P450 family 46 subfamily A member 1, Gfap: Glial fibrillary acidic protein, Hmgcr: 3-hydroxy-3-methylglutaryl-CoA reductase, Nr1h2: nuclear receptor subfamily 1 group H member 2, Nr1h3: nuclear receptor subfamily 1 group H member 3, Pou4f1: POU Domain, Class 4, Transcription Factor 1, Pou4f2: POU Domain, Class 4, Transcription Factor 2, Rbpms: RNA-binding protein with multiple splicing, Scarb1: scavenger receptor class B member 1, Srebf2: Sterol regulatory element-binding transcription factor 2, Thy1: Thymocyte differentiation antigen 1, Tnf: Tumor necrosis factor, Tradd: Tumor necrosis factor receptor type 1-associated death domain protein. (DOCX) [file pone.0264787.s003.docx]

**S1 Table. References of TaqMan assays (Applied Bioscience) used for QRTPCR analyses**

| Genes | Coding for | TaqMan assay references | Amplicon lenght |
| --- | --- | --- | --- |
| Abca1 | ABCA1 | Rn00710172_m1 | 76 |
| Apoe | ApoE | Rn00593680_m1 | 99 |
| Cd68 | CD68 | Rn01495634_g1 | 62 |
| Cyp27a1 | CYP27A1 | Rn01401086_m1 | 94 |
| Cyp46a1 | CYP46A1 | Rn01430188_m1 | 72 |
| Gfap | GFAP | Rn01253033_m1 | 75 |
| Hmgcr | HMGCR | Rn00565598_m1 | 71 |
| Ldlr | LDLR | Rn00598442_m1 | 76 |
| Nr1h2 | LXRβ | Rn00581178_m1 | 75 |
| Nr1h3 | LXRα | Rn00581185_m1 | 90 |
| Pou4f1 | Brn3a | Rn01465571_m1 | 102 |
| Pou4f2 | Brn3b | Rn01431271_g1 | 55 |
| Rbpms | RBPMS | Rn01478548_m1 | 56 |
| Scarb1 | SR-BI | Rn00580588_m1 | 75 |
| Srebf2 | SREBP2 | Rn01502638_m1 | 61 |
| Thy1 | Thy-1 | Rn00562048_m1 | 74 |
| Tnf | TNFα | Rn99999017_m1 | 108 |
| Tradd | TRADD | Rn01432142_g1 | 70 |

*^ApoE: Apolipoprotein E, Cd68: Cluster of Differentiation 68, Cyp27a1: cytochrome P450 family 27 subfamily A member 1, Cyp46a1: cytochrome P450 family 46 subfamily A member 1, Gfap: Glial fibrillary acidic protein, Hmgcr: 3-hydroxy-3-methylglutaryl-CoA reductase, Nr1h2: nuclear receptor subfamily 1 group H member 2, Nr1h3: nuclear receptor subfamily 1 group H member 3, Pou4f1: POU Domain, Class 4, Transcription Factor 1, Pou4f2: POU Domain, Class 4, Transcription Factor 2, Rbpms: RNA-binding protein with multiple splicing, Scarb1: scavenger receptor class B member 1, Srebf2: Sterol regulatory element-binding transcription factor 2, Thy1: Thymocyte differentiation antigen 1, Tnf: Tumor necrosis factor, Tradd: Tumor necrosis factor receptor type 1-associated death domain protein.^*
