# Supplementary material for: Retinal cholesterol metabolism is perturbated in response to experimental glaucoma in the rat
Source: PLoS One. 2022 Mar 11;17(3):e0264787. doi: 10.1371/journal.pone.0264787 (PMC8916636; doi:10.1371/journal.pone.0264787)
Supplement: S1 Raw images — (PDF) [file pone.0264787.s004.pdf]

18 hours post-laser

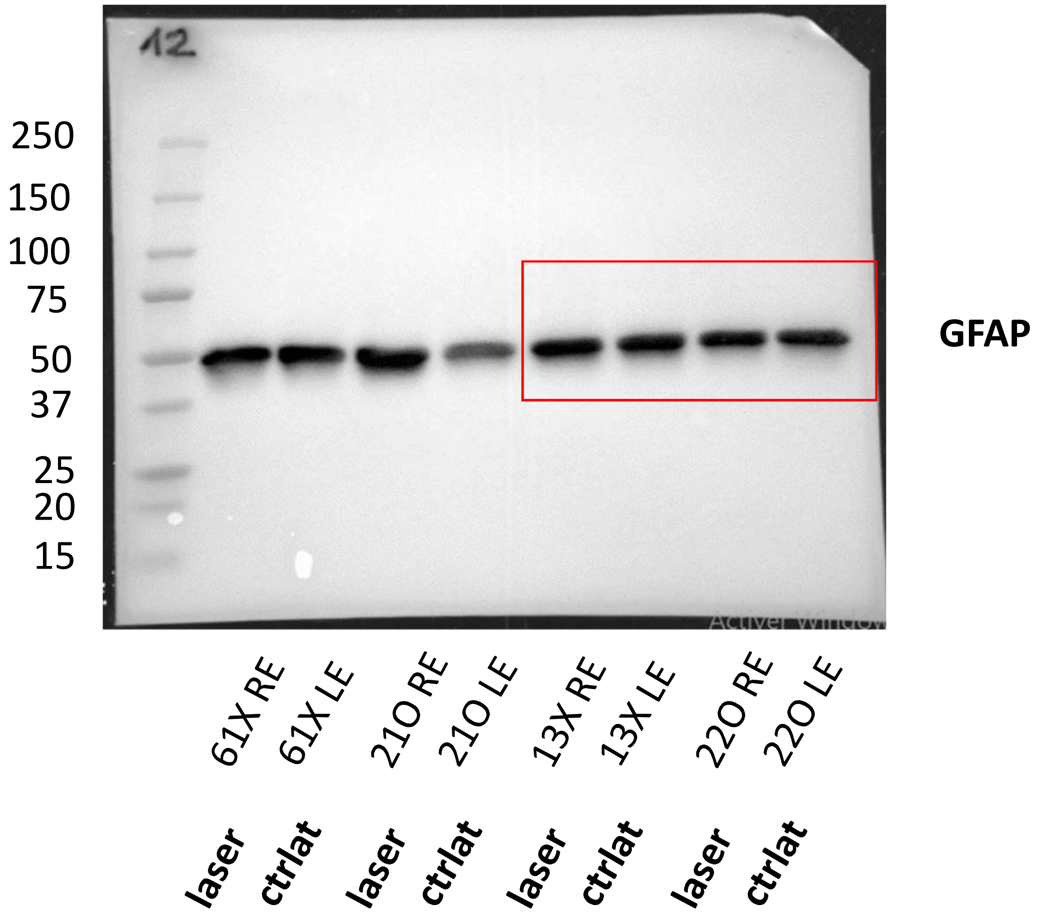

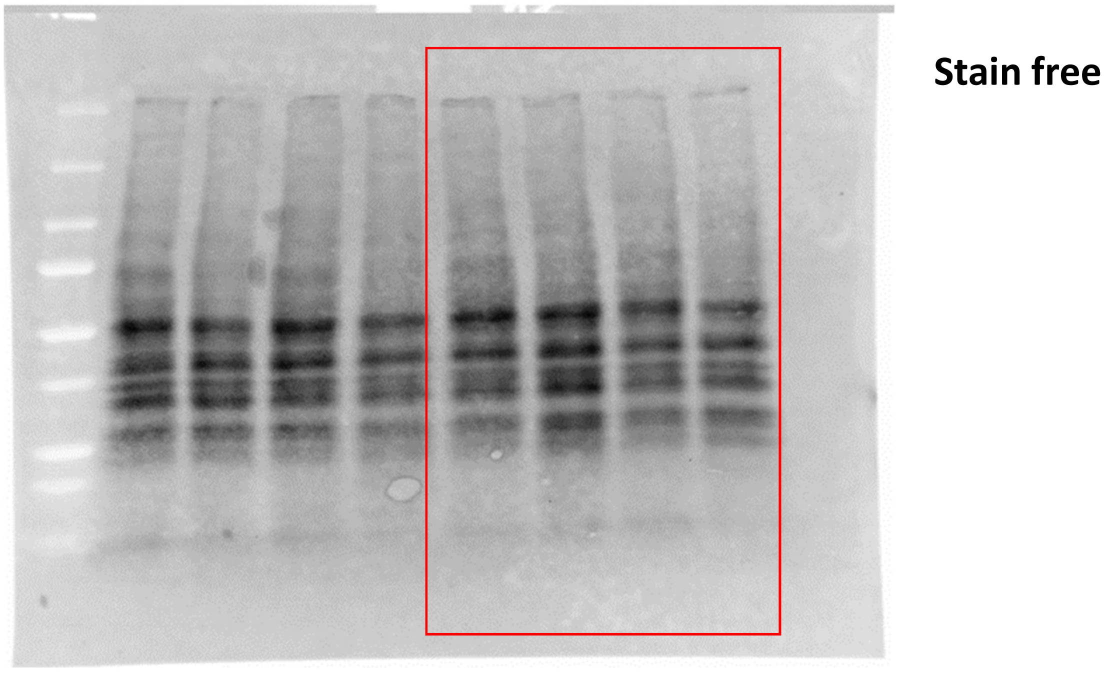

Used for illustration in Figure 2.E (18h panel) flipped right to left

RE: right eye (laser-treated)
LE: left eye (contralateral)

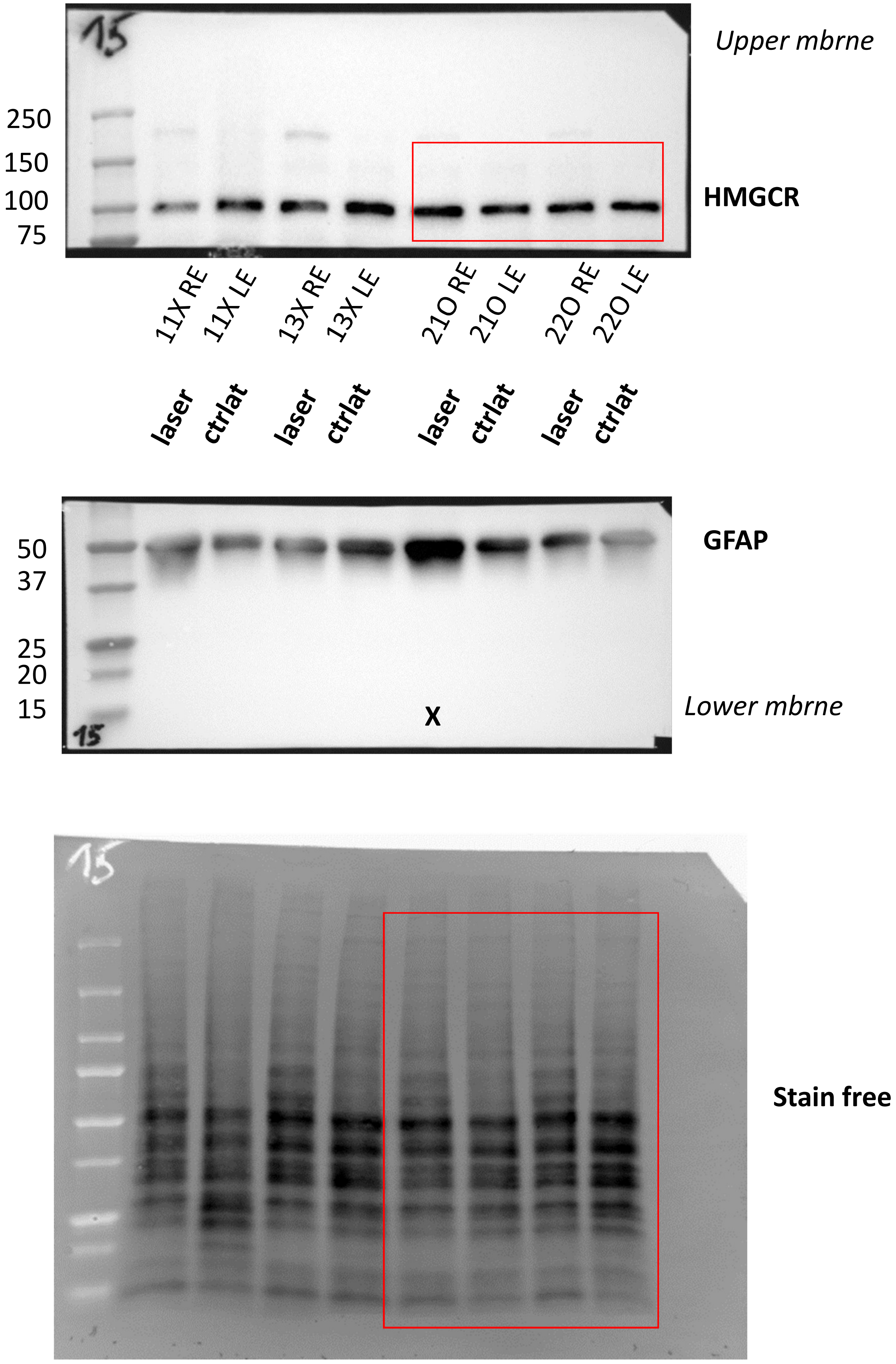

Used for illustration in Figure 5 (18h panel) flipped right to left

X: not included in the results

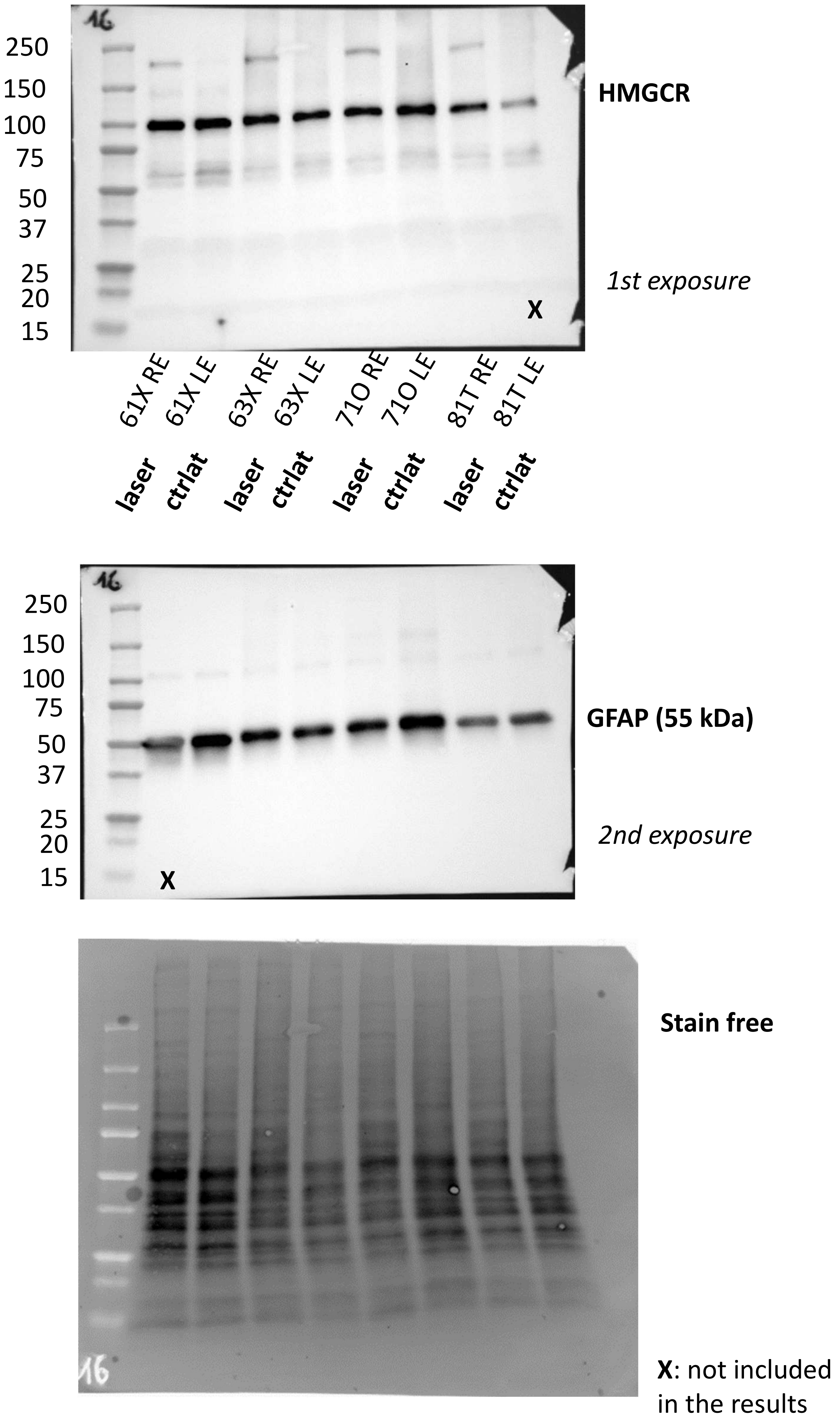

250
150
100
75
50
37
25
20
15
HMGCR
1st exposure
X
61X RE
61X LE
63X RE
63X LE
71O RE
71O LE
81T RE
81T LE
laser
ctrlat
laser
ctrlat
laser
ctrlat
laser
ctrlat
250
150
100
75
50
37
25
20
15
GFAP (55 kDa)
2nd exposure
X
Stain free
X: not included in the results

3 days post-laser

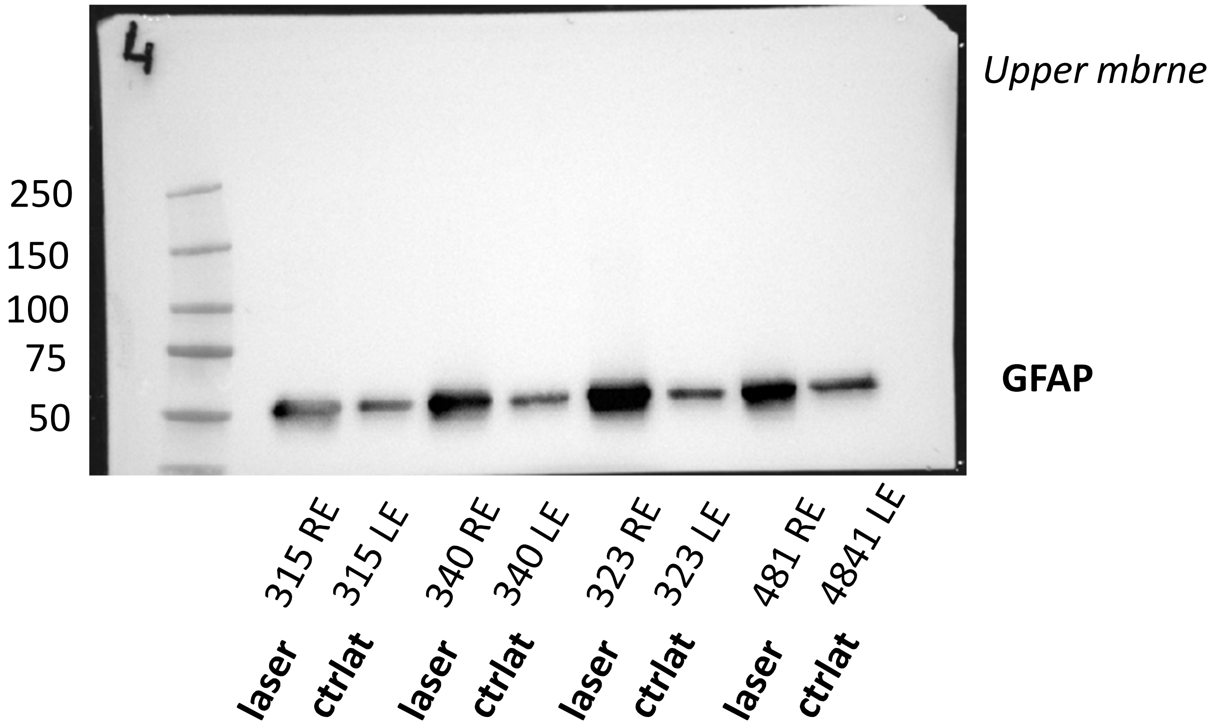

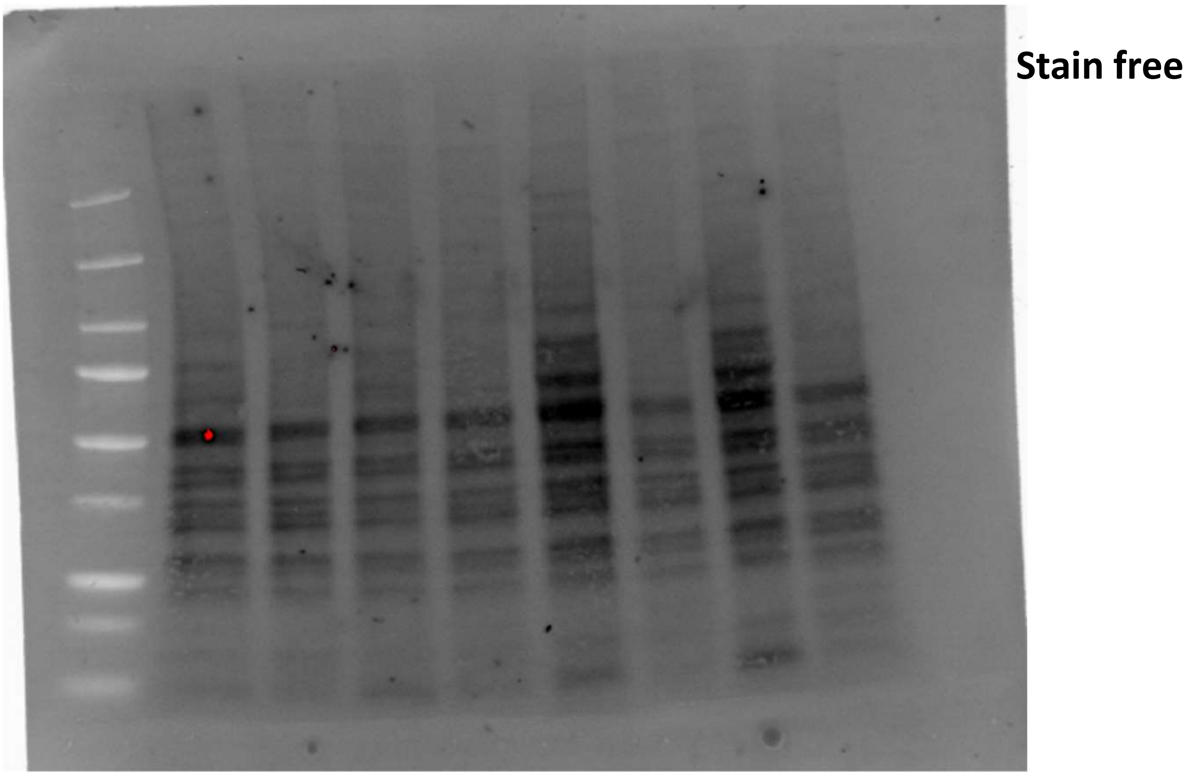

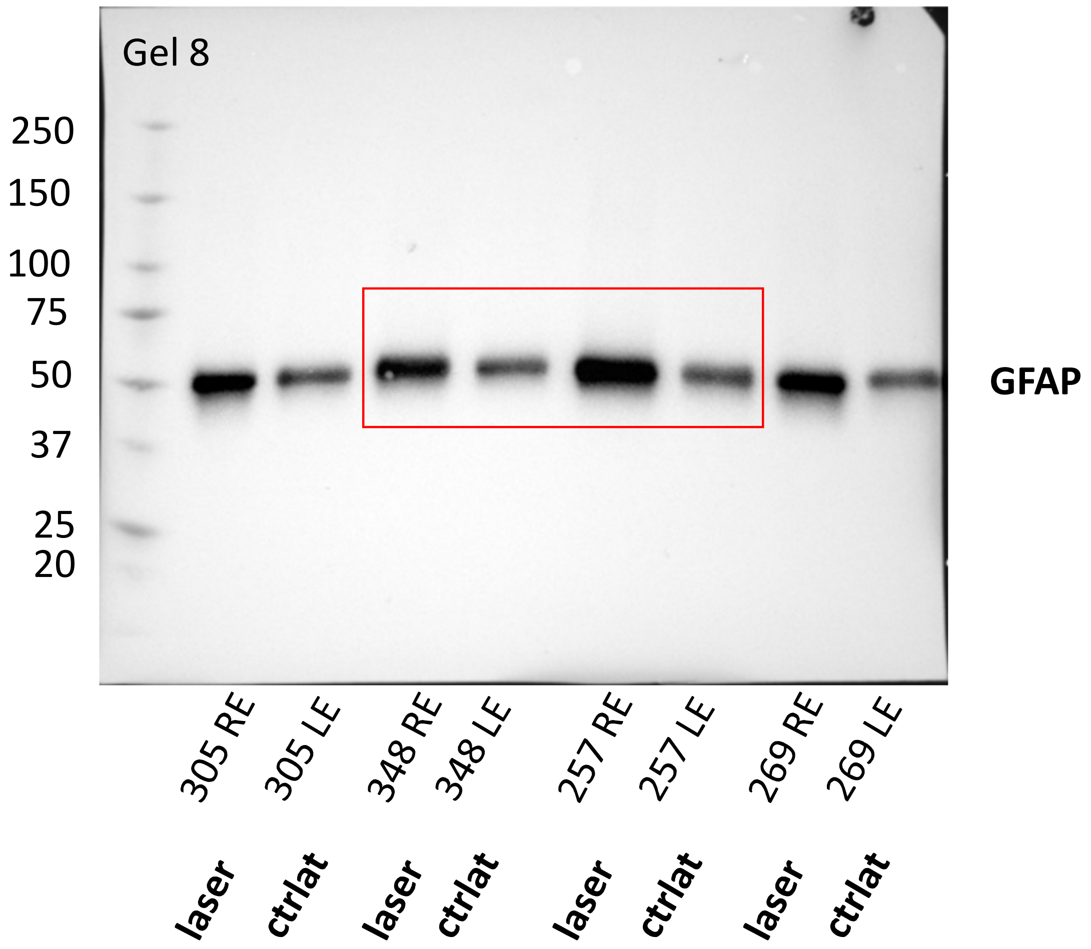

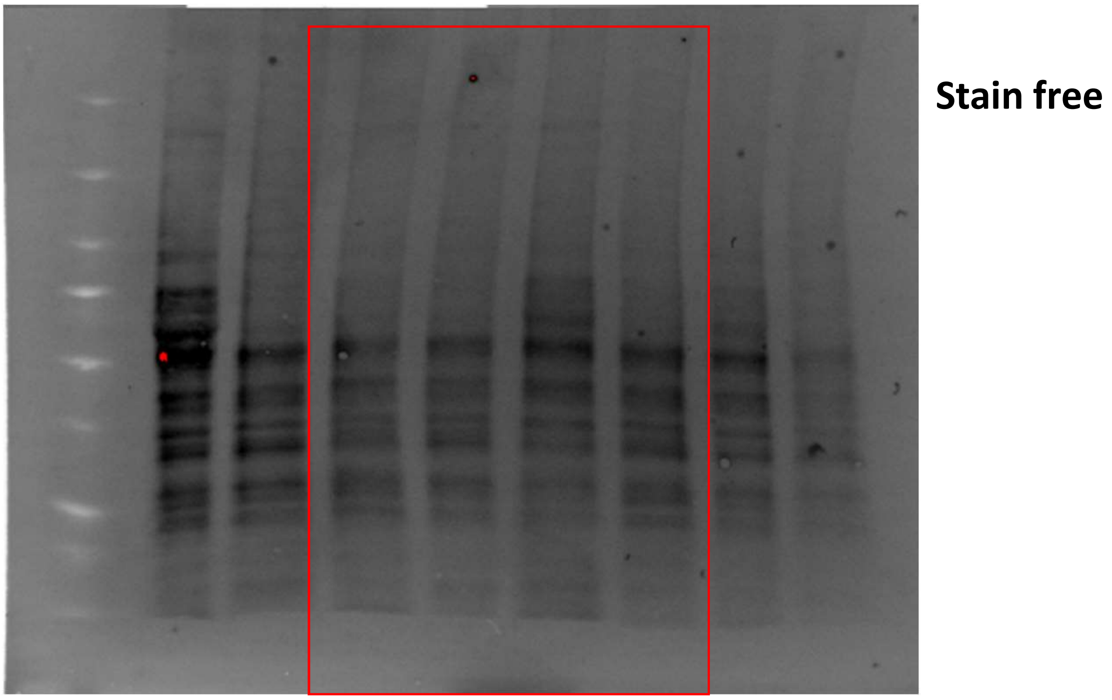

Used for Figure 2.E (3d panel) flipped right to left

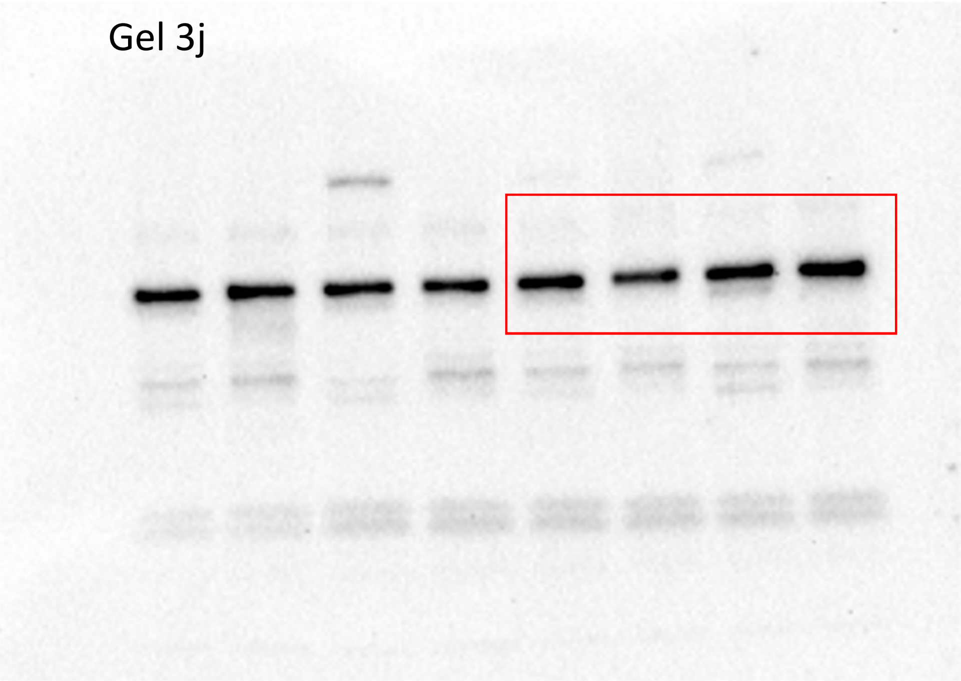

HMGCR

348 RE | 348 LE | 305 RE | 305 LE | 269 RE | 269 LE | 257 RE | 257 LE

laser | **ctrlat** | **laser** | **ctrlat** | **laser** | **ctrlat** | **laser** | **ctrlat**

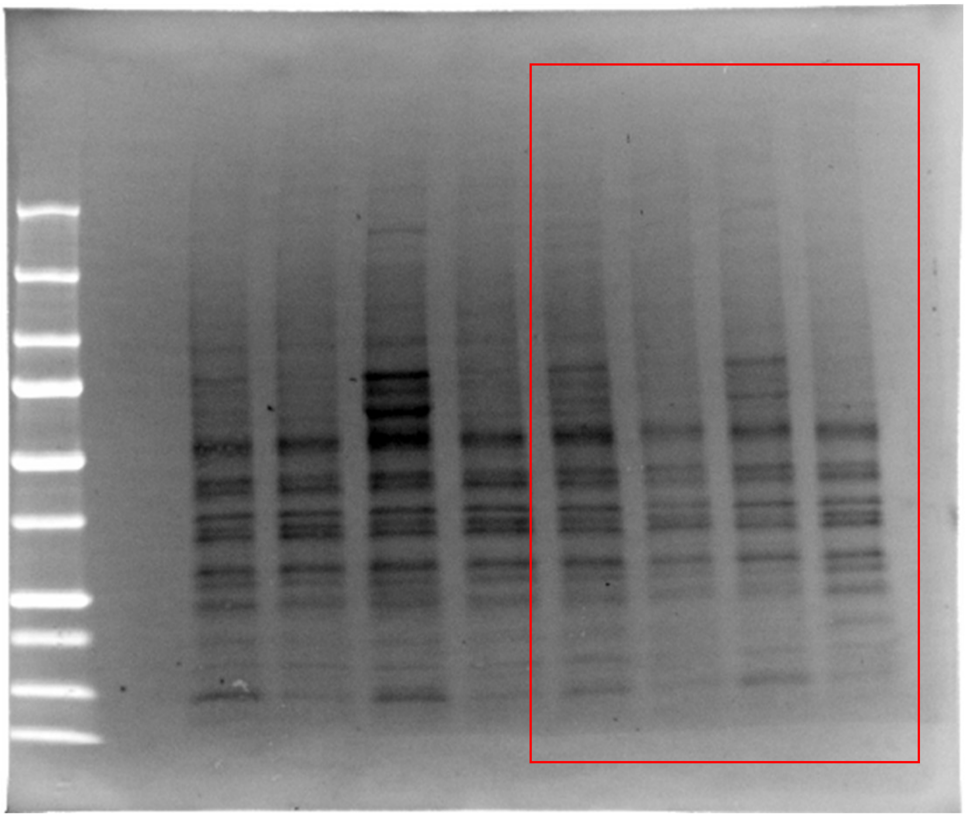

Stain free

Used for illustration in Figure 5
(3d panel) flipped right to left

1 month post-laser

Upper mbrne

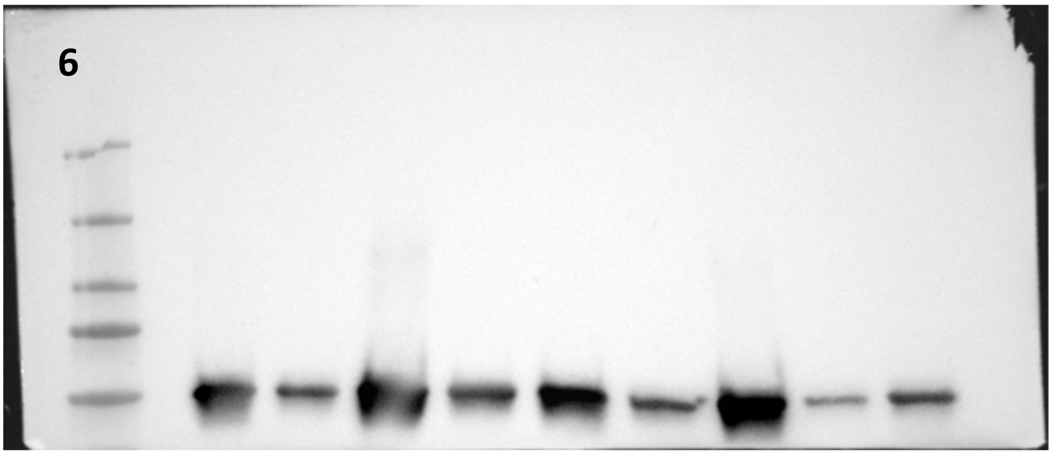

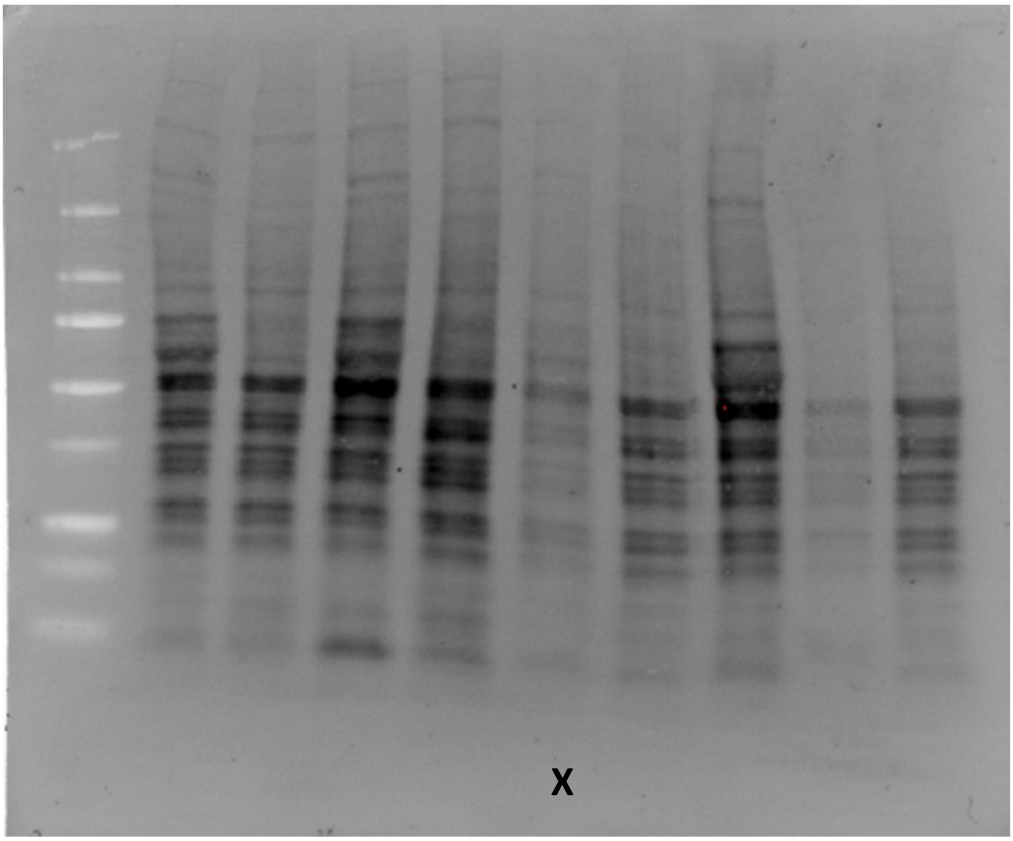

X: not included
in the results

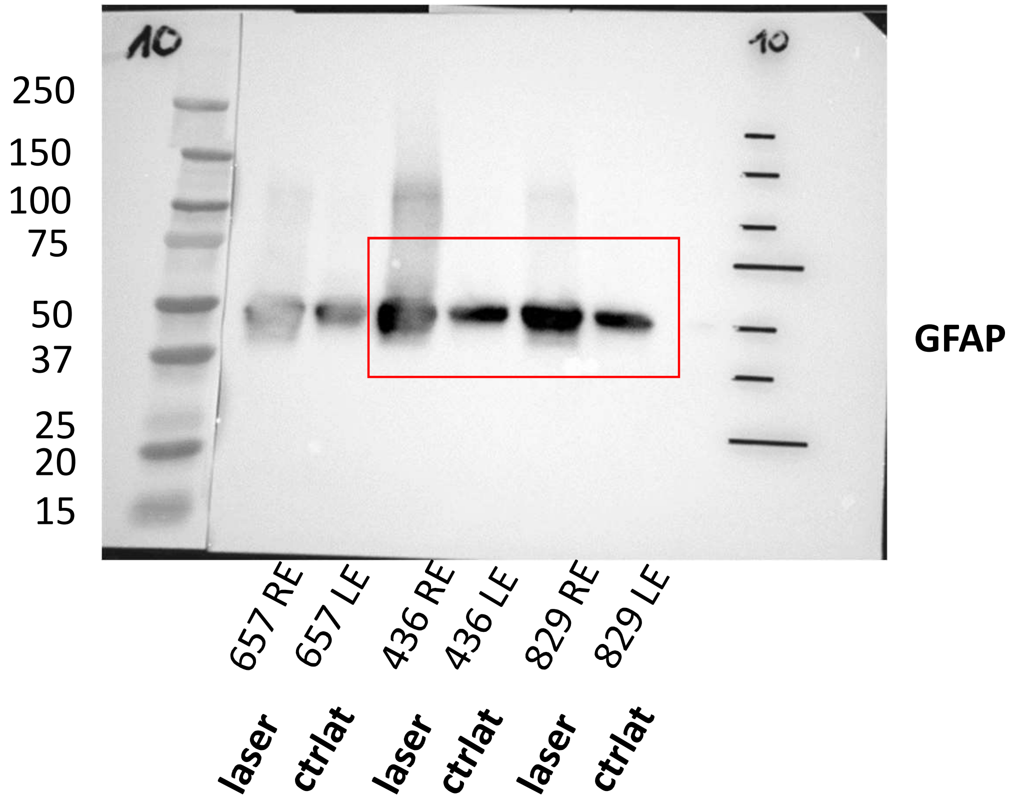

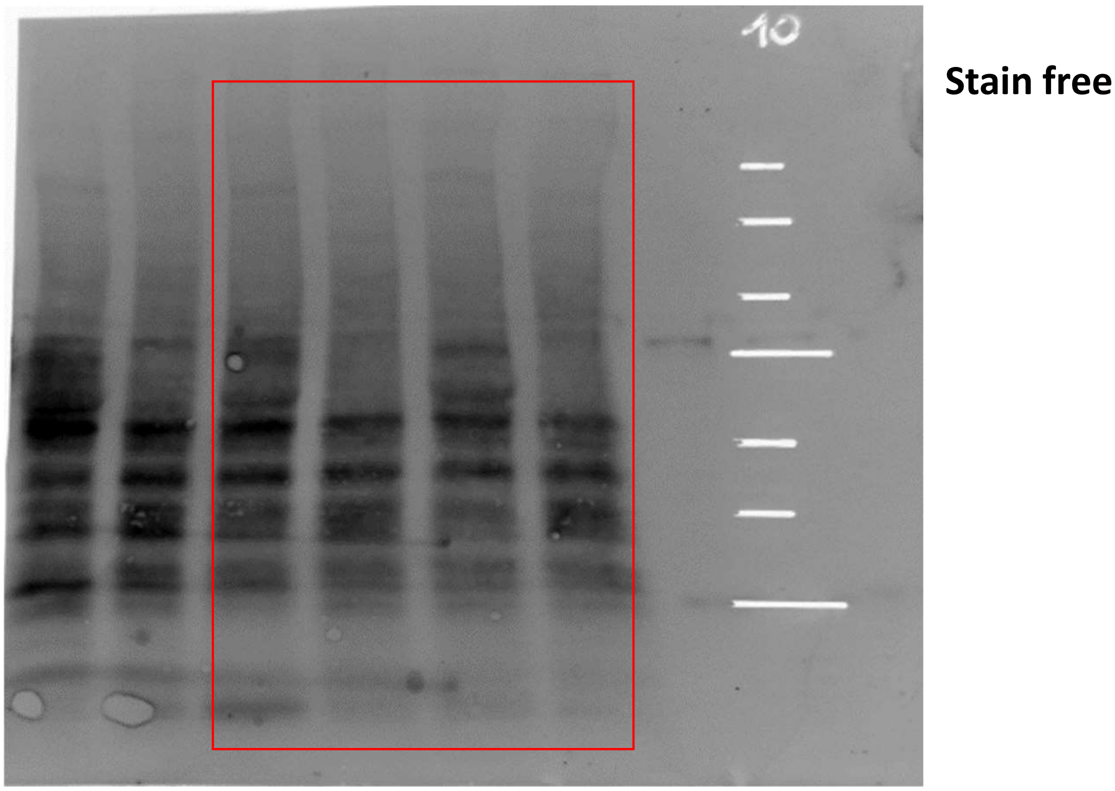

Used for Figure 2.E (1m panel) flipped right to left

NB: the dual color marker was mistakenly used instead of the all blue marker. It had to be cut for the stain free exposure because of signal interference. It was replaced at its proper position for the GFAP exposure

Gel 1m

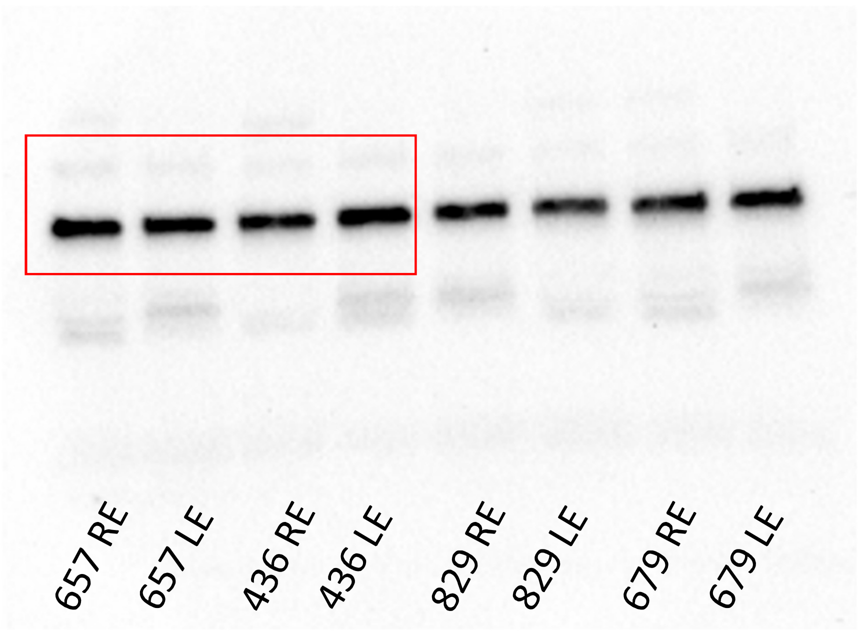

Stain free

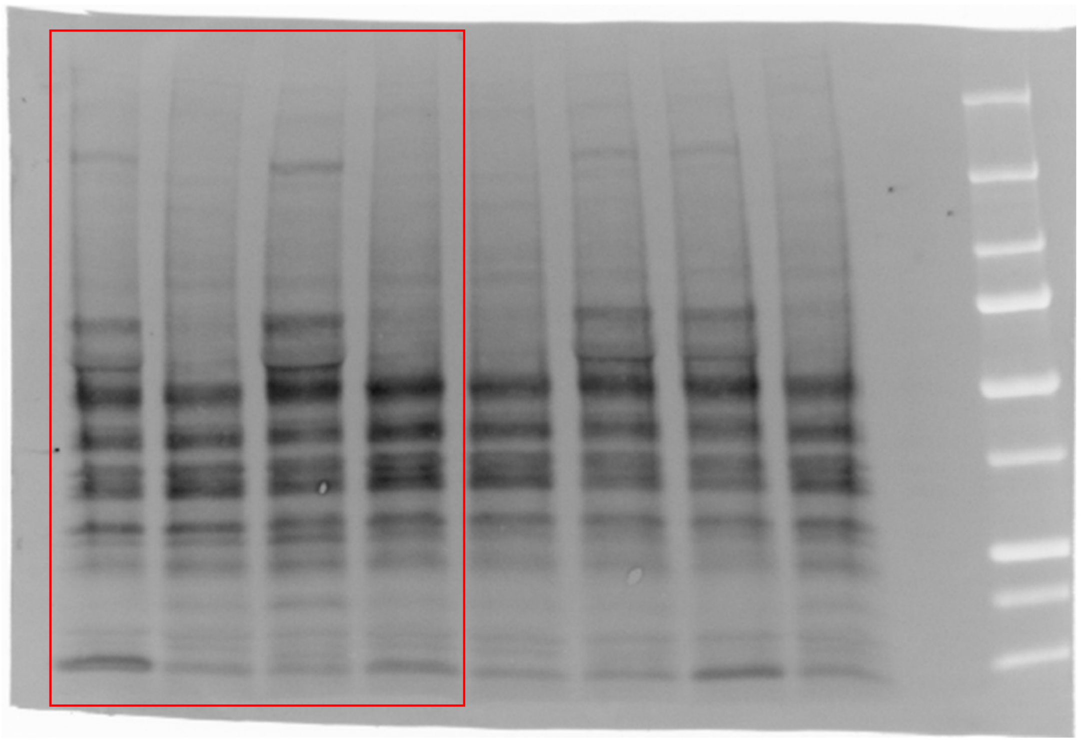

Used for illustration in Figure 5
(1m panel) flipped right to left
