# Supplementary material for: Retinal cholesterol metabolism is perturbated in response to experimental glaucoma in the rat
Source: PLoS One. 2022 Mar 11;17(3):e0264787. doi: 10.1371/journal.pone.0264787 (PMC8916636; doi:10.1371/journal.pone.0264787)
Supplement: Attachment — Submitted filename: Response to Reviewers.docx [file pone.0264787.s005.docx]

PONE-D-21-27664

Retinal cholesterol metabolism is perturbated in response to experimental glaucoma in the rat

PLOS ONE

5. Review Comments to the Author

Please use the space provided to explain your answers to the questions above. You may also include additional comments for the author, including concerns about dual publication, research ethics, or publication ethics. (Please upload your review as an attachment if it exceeds 20,000 characters)

Reviewer #1: The authors have characterized cholesterol metabolism perturbations in a rat laser-induced glaucoma model. The manuscript is well written, and the study provides valuable information about retinal metabolic changes resulting from elevated pressure (in the rodent model at least).
The methods are well described, and the techniques are all well-established in the literature.
The dynamic changes in cholesterol metabolism have been well demonstrated. The reduced expression of Cyp27a1, in response to elevated IOP treatment, but not Cyp46a1, is a most interesting finding.

The authors provide a thoughtful discussion of the possible underlying mechanisms.
Conclusions are presented in an appropriate fashion and are supported by the data.
The Figures are of good quality and the schematics helpful.

Specific queries

There is one very curious result that needs some explanation

The authors have measured RGC injury using a combination of RGC-specific mRNA quantification with qPCR and with RGC counts using Brn3a-immunostained retinal flat mounts.

Fig 3A shows early loss of Brn3a message with recovery by 1 month. The RBPMS and Thy-1 RQ means also recover.

Curiously, the flat mount data (Fig 3C) show a relatively modest but increasing loss of Brn3a immunoreactive cells out to 1 month.

How is the mRNA data at 1 month reconciled with the flat mount data?

We thank reviewer #1 for his interest in our study and his appreciation of the manuscript. We agree that the recovery of the gene expression of RGC specific markers is most intriguing. The hypothetical explanation we can propose is that the expected decrease of qPCR signal due to the loss of RGC, as measured on flatmounted retinas at 1 month post-laser, was compensated by an overexpression of these same markers by the remaining RGC or other cell types in the retina. We added this point to the corresponding Result section (p 16).

Reviewer #2: The paper „Retinal cholesterol metabolism is perturbated in response to experimental glaucoma in the rat“ is an intersting topic. In conclusion, they report that ocular hypertension is associated with transient major dynamic changes in retinal cholesterol metabolism.
However, it is not clearly written and confusing as many results are shown such as Iba-1 , GFAP , Caspase changes, that don`t have much to do with the cholesterol changes. It is not even explained how they could be associated with those changes. The findings they had regarding cholesterol metabolism were only „As early as 18 hours after the laser procedure, genes implicated in cholesterol biosynthesis and uptake were upregulated (+49 % and +100 % for Hmg-CoA reductase and Ldlr genes respectively, vs. naive eyes) while genes involved in efflux were downregulated (-26 % and -37 % for Apoe and Cyp27a1 genes, respectively). „ No furhter validation of those changes was done , e.g. IHC, Proteoics, WB or no furhter explanation oft he pathomechanims. All other findings are descriptive and the authors fail to explain the association.
It is a shame as the storyline is not obvious and loads of findings are reported which might be of interesting value but the association is not clear.

We thank reviewer #2 for his interesting comments on our manuscript and acknowledge the lack of clarity and explanation of the physiopathologic mechanisms.

Reviewer #2 raises the fact that many results don’t have much to do with the cholesterol changes. Indeed, the results shown in Figures 2 (gliosis and inflammation) and 3 (RGC death) aim to characterize our experimental model of glaucoma. We presented these results in order to reveal the potential triggers and/or consequences of the changes in cholesterol metabolism we report in this specific model. We changed the title of the corresponding Result section to “characterization of the experimental model of glaucoma” to clarify the utility of these data (p 14).

The lack of explanation of the pathomechanisms is also a matter of criticism. At that stage of the study, we cannot explain the association between the increase in IOP, inflammation and gliosis we observed and the changes in cholesterol metabolism we measured. It is an exploratory work since no similar study had been performed so far. We made several hypotheses in the discussion section based on literature data to explain the potential link between cholesterol metabolism and i) increased IOP (lines 397-410), ii) inflammation and gliosis (lines 410-423), iii) RGC death (424-430). To make this clearer, we designed a scheme with a legend (see below). In this scheme, we also illustrate the idea that the restoration of cholesterol homeostasis might be protective against RGC death. If reviewer #2 considers it might be useful for the readers and not too speculative, we could include it to the final version of the manuscript, possibly in replacement of Figure 7. The abstract was also modified to make the potential association between the different observations clearer.

**Fig.8**. **A proposed model for the potential role of retinal cholesterol metabolism in our ocular hypertensive model of glaucoma.** The laser procedure induced increased IOP, Inflammation and gliosis. Consecutively, the activation of hypercholesterogenic mechanisms resulted in transient retinal hypercholesterolemia known to be toxic for neurons. Activation of hypocholesterogenic feed back mechanisms then enabled the restoration of cholesterol homeostasis and prevented more pronounced RGC death. Arrows represent the potential causal links between the events based on our observations and on commonly accepted pathways. Dashed arrows correspond to undetermined mechanisms.

Regarding the absence of validation of the measured changes in gene expression by measuring protein expression, we agree with reviewer #2 it is a limitation of our work. We did it for HMGCR using Western Blot (Fig. 5) and did not measure any change in protein expression despite the significant changes in gene expression as measured by RT-qPCR, maybe due to post-translational regulations. We also investigated the 2 other players whose gene expression was strongly regulated: ABCA1 and LDLR. Unfortunately, the antibodies tested under various conditions generated blots of poor quality that we considered insufficient to provide reliable results. As one can see below, the signal of bands at the expected molecular weight was faint while we detected many aspecific bands. However, the coordinated regulation of gene expression we observed at the different time points combined with a coherent modification in sterol levels gives us confidence in the fact that a regulation of cholesterol metabolism is actually involved in our experimental model of glaucoma.
